# Prevalence and reasons for non‐nursing tasks as perceived by nurses: Findings from a large cross‐sectional study

**DOI:** 10.1111/jonm.13451

**Published:** 2021-08-31

**Authors:** Silvia Grosso, Jessica Longhini, Saverio Tonet, Ines Bernard, Jacopo Corso, Denis de Marchi, Laura Dorigo, Gianluca Funes, Massimo Lussu, Nicolas Oppio, Luca Grassetti, Luigi Pais Dei Mori, Alvisa Palese

**Affiliations:** ^1^ Ordine delle Professioni Infermieristiche Belluno Italy; ^2^ Department of Medical Sciences University of Udine Udine Italy

**Keywords:** clinical competence, interprofessional work, non‐nursing task, nursing, nursing staff

## Abstract

**Aim:**

The aim of this study is to describe the prevalence and reasons for non‐nursing tasks as perceived by nurses.

**Background:**

Four types of non‐nursing tasks have been identified to date: (a) auxiliary; (b) administrative, (c) expected by allied health care professionals; and (d) medical. However, no studies on a large scale have been performed with the aim of identifying the prevalence of all of these non‐nursing tasks, and factors promoting or hindering their occurrence, given that they represent a clear waste of nurses' time.

**Methods:**

A cross‐sectional study in 2017, following The Strengthening the Reporting of Observational studies. All active nurses registered in an Italian provincial Nursing Board (=1331) willing to participate were involved. A questionnaire survey exploring the nature of the nursing tasks performed in daily practice and the underlying reasons was administered via paper/pencil and e‐mail.

**Results:**

A total of 733 nurses participated of which 94.5% performed at least one type of non‐nursing task, mainly administrative and auxiliary. Auxiliary tasks are less likely among nurses working in a community (odds ratio [OR] 0.43, 95% CI 0.29–0.63, *p* < .01) or in a residential (OR 0.41, 95% CI 0.23–0.72, *p* < .01) setting, in critical (OR 0.29, 95% CI 0.16–0.54, *p* < .01) or surgical (OR 0.37, 95% CI 0.19–0.75, *p* < .01) hospital settings, and when they deal with unexpected clinical events (OR 0.58, 95% CI 0.44–0.77, *p* < .01). Greater adequacy of nursing resources decreases the occurrence of auxiliary tasks (OR 0.98, 95% CI 0.97–0.99, *p* < .01), whereas the need to compensate for a lack of resources (OR 1.44, 95% CI 1.07–1.93, *p* < .01) increases it.

**Conclusions:**

Around one‐third of shift time is devoted to non‐nursing tasks; working in a hospital, in medical units, with lack of resources and with patients with predictable clinical conditions might increase the occurrence of auxiliary tasks.

**Implications for nursing management:**

Strategies to increase the time available for nursing care should consider the type of tasks performed by nurses, their antecedents and the value added to care in terms of patient' benefits.

## 1 BACKGROUND

The concept of non‐nursing tasks, first identified in 1961 (Connor, 1961), is attracting new interest among researchers as these represent from 35% (Fitzgerald et al., 2003) to 62% of the nursing shift duration (Bruyneel et al., 2013) and carry negative consequences for both patients and nurses (e.g., Bekker et al., 2015). To our best knowledge, the “non‐nursing tasks” term has been first used by Aiken et al. (2001) to indicate activities not requiring nursing education that nurses have to perform. Bruyneel et al. (2013), some years later, have described non‐nursing tasks as activities enacted by nurses ‘below their skill level’ (Bruyneel et al., 2013). Cleaning rooms, delivering or retrieving food trays, escorting patients and performing auxiliary services have all been reported as examples of non‐nursing tasks (Aiken et al., 2001; Bruyneel et al., 2013). However, alongside these tasks, nurses have been reported to perform also administrative tasks, such as replenishing charts and forms, answering phone calls and planning appointments (e.g., Hendrich et al., 2008).

Over the years, the meaning of the term “non‐nursing tasks” has been expanded to activities belonging to allied health care professionals—that is, other professionals excluding physicians, dentists or nurses (Featherston et al., 2020). Mobilizing patients on Sunday when physiotherapists are absent (Grosso et al., 2019) has been reported as an example of these non‐nursing tasks. Moreover, nurses perform also tasks failing in the scope of the medical discipline, such as making decisions about diagnostic procedures when physicians are unavailable at the bedside (Grosso et al., 2019). As a result, four main types of non‐nursing tasks have been documented to date: (a) tasks with an administrative nature; (b) auxiliary tasks meant as those that could be delegated to nurses' aides, assistants and unlicensed health workers; (c) tasks belonging to the scope of practice of allied health care professionals; and (d) tasks from the medical profession.

Non‐nursing tasks have become more frequent in the last decades due to spending reviews and cost‐cutting measures, both of which have increased the flexibility required from nurses (Scott et al., 2013). Changes in the staff mix and reductions in the number of nurses' aide have also increased the occurrence of non‐nursing tasks. For example, when units are understaffed for housekeepers and porters, their tasks are expected to be performed by nurses (Kearney et al., 2016) or by nursing students who might learn that it is ‘normal’ to perform these tasks, thus perpetuating the phenomenon (Palese, Ambrosi, et al., 2019).

Nurses are called to be flexible in performing a range of interventions outside the scope of their education and practice, substantially eroding the care offered, leaving patients' needs unmet. Missed nursing care (e.g., educating patients and monitoring vital signs) (Al‐Kandari & Thomas, 2009), nurses' perceptions of wasting time (Hendrich et al., 2008), burnout (Tunc & Kutanis, 2009) and job dissatisfaction (Bekker et al., 2015) have been reported as consequences of non‐nursing tasks.

Despite the recognized relevance, differences across countries on the scope of nursing education and practice and the heterogeneous perceptions among nurses with regard to what nursing care is and is not, as well as the continuous development of the nursing role (Benton et al., 2017), still prevent a full understanding of the factors promoting or hindering the occurrence of the phenomenon. Moreover, research available has focused mainly on those activities delegable to nurses' aides, assistants and unlicensed health workers (Hewko et al., 2015; Palese, Gnech, et al., 2019). However, to our best knowledge, no studies have explored antecedents of non‐nursing tasks as perceived by nurses, thus preventing the full identification of interventions aimed at minimizing the occurrence of the phenomenon and its negative consequences.

Therefore, with the aim of improving the knowledge in the field, the principal purpose of this study was to describe the prevalence of the four different types of non‐nursing tasks (auxiliary, administrative, that of allied health care professionals and medical); the secondary aim was to explore the reasons of auxiliary tasks as perceived by nurses. The underlying reasons have been explored in more depth to increase the available evidence for nurse managers to consider what tasks add value to nursing care. Value‐added nursing care has been defined as those activities benefitting patients' outcomes and their experience, such as performing admission and discharges; teaching and/or supporting patients, family and caregivers; reviewing clinical charts; or performing direct care at the bedside (Dearmon et al., 2013). Differently, nonvalue‐added activities have been reported to consist, for example, in searching for and retrieving equipment, in escorting patients, doing paperwork and delivering supplies (Upenieks et al., 2007). Furthermore, we considered that (a) administrative tasks might reflect part of nursing care processes (e.g., programming a care pathway), (b) that of allied health care professionals might be part of the nursing scope of practice in some contexts (e.g., rehabilitation units) and (c) medical tasks might express an advancement of the profession in some fields (e.g., critical care).

## 2 METHODS

### 2.1 Study design and setting

A cross‐sectional study design has been performed in 2017 and reported here according to The Strengthening the Reporting of Observational studies in Epidemiology studies checklist for cross‐sectional studies (von Elm et al., 2008) (Table S1). The study has been performed in an Italian province of 199,802 inhabitants, extending over a mountainous territory (3610.20 km^2^) in the eastern Alps sector, where most Dolomite groups are present.

### 2.2 Sample

Eligible nurses were (a) registered in the Nursing Board of Belluno (Ordine delle Professioni Infermieristiche, Belluno, Italy), (b) working at the time of the survey and (c) were willing to participate in the study. From the list of 1987 nurses registered, 1331 nurses were deemed eligible.

### 2.3 Variables and data collection instrument

A questionnaire survey was developed by 11 members of the Nursing Board (advanced educated and with a range of role responsibilities, from clinical to managerial) during five consecutive meetings, each of them 1.5 h in length. In these meetings, a review of the literature (e.g., Biondino, 2017; Bruyneel et al., 2013; D'Angelo, 2014; Grosso et al., 2019; Gussoni, 2016; Kearney et al., 2016; Lu et al., 2008; McKenna, 1998) was performed. Also, 64 letters written by nurses to the President of the Nursing Board requesting help/advice with regard to non‐nursing tasks were analysed. The questionnaire survey was piloted in a subgroup of 30 nurses, and no changes were required: in its final version, the survey included three data collection areas:

- Non‐nursing tasks end point. In this section, participants were asked to indicate if they performed activities (yes/no) included in one or more non‐nursing task category (auxiliary, administrative, that of allied health care professionals and medical tasks). In order to ensure accuracy, for each non‐nursing task category, brief examples were provided based on the literature (Aiken et al., 2001; Bruyneel et al., 2013; Dearmon et al., 2013; Grosso et al., 2019; Upenieks et al., 2007) and Italian laws:
  ○ Administrative tasks as replenishing charts and forms, answering phone calls and planning appointments, scheduling meetings not regarding patients, as a secretary;
  ○ Auxiliary tasks, as cleaning rooms, delivering or retrieving food trays, escorting patients, performing auxiliary services, searching for and retrieving equipment;
  ○ Allied health professional tasks, as mobilizing the patient during the weekend (physiotherapist), providing nutritional advice (dietician), foot care (podologist), cognitive or behavioural rehabilitation (professional educator, psychiatric technician); and
  ○ Medical tasks, as prescribing medications or diagnostic examinations, not allowed to be performed by nurses according to the Italian law.
    For each non‐nursing task category performed by nurses, they were also asked to indicate: (a) how often had they performed each during the last shift (from 1, *never*, to 4, *always*), (b) in which shifts these occurred more often (e.g., morning) and (c) the amount of time dedicated to each non‐nursing staff during a shift (in percentage, up to 100%).

- Explanatory variables: three levels of data have been collected:
  1. Demographic (e.g., age and gender).
  2. Professional, such as education (e.g., diploma or bachelor and advanced education or not); experience as a nurse and as a nurse in the unit (years); workplace (hospital and community) and unit (medical, surgical) where nurses were working at the time of the survey; weekly working hours, overtime accumulated in the last 3 months (as paid or not paid according to the trust rules) and shift profile (shift, daily, morning or night worker); patients taken care during the last shift, admitted and discharged (number); adequacy of the nursing resources perceived (from 0% *never* to 100% *all the time* of the shift) and the model of nursing care delivery used in the unit, namely, (a) functional nursing: nurses perform assigned tasks to all patients in the unit in a given time; (b) team nursing: a team composed by nurses and nurses' aides, work together under the guide of a nurse team leader to provide care to a group of patients or (c) other, as mixed models (functional and team). Moreover, the degree of satisfaction in the role, as a nurse and in the working group (from 1, *never*, to 4, *always*), as well as the intention to leave the unit (yes/no) was also investigated.

- Reasons for non‐nursing tasks: nurses were asked to report the perceived reasons (Likert scale = 1, *not a reason*, to 4, *a significant reason*). The validity of the items was assessed by an exploratory factor analysis (EFA) and a confirmatory factorial analysis (CFA) by randomizing the database in two sub‐datasets (245 questionnaires/each after missed items removed). According to the factor loadings (>.350) of the EFA, the 14 items retained were categorized into four factors explaining a total variance of 67.02% (Cronbach's [*α*] = .867) (Table S2):
  - ‘Compensating the lack of resources’ (11 items, variance = 20.78%, *α* = .796), reflecting the need of nurses to provide several tasks out of the scope of their practice due to the lack of human resources available at the unit level (examples of items: ‘Lack of nurses’ aides'; ‘Excessive workloads’);
  - ‘Being pressed by the organisational culture’ (3 items, variance = 17.63%, *α* = .811) expressing the organisational culture pressing nurses to perform non‐nursing tasks because it is expected of them to perform all activities and to be flexible (e.g., ‘Organizational routine’; ‘Rules established by the heads of the department/hospital’);
  - ‘Dealing with unexpected clinical events’ (3 items, variance = 15.0%, *α* = .767) expressing the increased workloads required to manage unpredictable clinical situations (e.g., ‘Unexpected critical patients/situations’; ‘High number of admissions’);
  - ‘Protecting patients’ (3 items, variance = 13.61%, *α* = .728), expressing the willingness of nurses to satisfy patients' needs by keeping a positive atmosphere in the team in circumstances where tasks are at risk of being left undone (e.g., ‘Ensure patients’ outcomes; ‘Ensure that all tasks required are carried out’).

At the CFA, the indexes confirmed a satisfactory fit for the model based on the following data: standardized root mean square residual = .069; root mean square error of approximation = .083; 90% confidence interval = .070–.095; comparative fit index = .893; Tucker–Lewis's index = .859; and minimum function test statistic = 1378.447; *p* < .001.

### 2.4 Data collection

The questionnaire survey was sent by e‐mail for those with an active e‐mail and administered via paper and pencil for nurses working in hospitals and nursing homes with no available e‐mail address. Nurses who received the online survey by e‐mail gave their written consent and then the survey was displayed and filled in. The remaining received the paper/pencil survey questionnaire in an envelope at the unit level; then, they filled in, and the survey questionnaire was collected in a closed box allocated in each unit.

### 2.5 Data analysis

Data collected were inserted in an excel database by two researchers and checked by a third researcher (see authors). Then, after having assessed the quality of the data and the missed items, descriptive and inferential statistics were performed. Categorical variables were reported as absolute and percentage frequencies, whereas continuous variables were expressed by means and 95% confidence of interval (CI). Explanatory variables were investigated in their differences, if any, between each group of nurses who performed the task under study (e.g., auxiliary tasks) and those who did not (Chi‐square [*χ*] and *t* tests).

Then, an evaluation of the appropriateness of the sample size was conducted: the sample met recommendations for statistical significance set at 5% using the statistical method known as structural modelling processes (Hair et al., 2014). Moreover, the database was checked to remove missing values (<1%) prior to employing the full information maximum likelihood approach (Arbuckle et al., 1996).

A path analysis model was estimated to detect which explanatory variables account for the variance of the auxiliary tasks. Multiple regressions have been performed considering both linear and generalized linear models. The outcome (=performing auxiliary tasks) was entered, while explanatory variables (Tarling, 2009) included were those (a) emerged in the bivariate analysis as significant (e.g., age; professional, for example, place of work, unit and models of care delivery), (b) documented in the literature (e.g., perceived reasons ‘Compensating the lack of resources’). Excluded from these variables were shifts (e.g., mornings) and the shift profile of nurses (e.g., shift workers) because these were not peculiar to auxiliary tasks, and because both were affected by the work unit (e.g., hospital versus community). On the other hand, the nurse's intention to leave and satisfaction were entered as antecedents of auxiliary tasks assuming that nurses awaiting to leave the unit and unsatisfied are less engaged professionally and more likely to perform these tasks.

Sequential multiple regression analyses then explored direct and indirect effects: the standardized coefficients beta (*β*) and odds ratio (OR, 95% CI) were estimated according to the nature of each variable. Standard errors (SEs), test statistics (*z* values), and *p* values (*P*[>|*z*|]) were also calculated to perform the inferential analysis (available from authors).

The SPSS Statistical Package version 26, the R Core Team (R Core Team, 2017) and the lavaan (Rosseel, 2012) package in R were used. The statistical significance was set at *p* < .05.

### 2.6 Ethical issues

The General Assembly of the Belluno Nursing Board (Ordine delle Professioni Infermieristiche, Belluno, Italy) and the Nursing Board Steering Committee approved the research project (n. 30, on 16.07.2015). International and national ethical principles have been fulfilled. Nurses were invited to participate on a voluntary basis, and no incentives were offered. They were fully informed about the study aims, and their informed consent was collected in the first page of the survey questionnaire. Hospitals, units and community settings were anonymized.

## 3 RESULTS

### 3.1 Participants

A total of 743 nurses out of 1331 (55.8%) agreed to participate, and 10 survey questionnaires (0.7%) were not completed. Therefore, 733 responses were considered valid for analysis.

Most participants were female (616, 84%), and the average age was 43.6 (95% CI 42.9–44.2) years. The majority had a nursing diploma (498, 67.9%), and a few of them have achieved an advanced nursing education (111, 15.1%). Most participants were employed in a hospital (599, 81.7%), mainly in medical (229, 31.2%) and critical care units (154, 21%); fewer nurses were working in community settings (59, 8%).

Participants worked for an average of 22 years (95% CI 21.3–22.7) and 12 (95% CI 11.4–12.7) in the current unit (Table 1). Most of them were working full time (578, 78.9%) as shift workers (373, 50.9%). The majority (533, 68.7%) worked overtime in the last 3 months, accumulating on average 25.6 h (95% CI 23.7–27.6).

**TABLE 1:** Participants profiles

Variables	Total *N* (%), average (95% CI)
Participants, *n*	733 (100)
Demographic variables	
Gender, *n*	
Female	616 (84.0)
Male	117 (16.0)
Age, years	43.6 (42.9–44.2)
Professional variables	
Nursing education, *n*	
Nursing Diploma	498 (67.9)
Nursing Bachelor	196 (26.7)
Nursing Diploma + Bachelor	39 (5.3)
Advanced educated, *n*	111 (15.1)
Experience as a nurse, years	22.0 (21.3–22.7)
Experience in the setting, years	12.0 (11.4–12.7)
Working at, *n*	
Hospital	599 (81.7)
Community	59 (8.0)
Residential	51 (7.0)
Freelance	15 (2.0)
Other	9 (1.2)
Setting, *n*	
Medical	229 (31.2)
Critical care	154 (21.0)
Surgical	134 (18.3)
Maternal/paediatrics care	34 (4.6)
Home care	69 (9.4)
Nursing home	54 (7.4)
Other	59 (8.0)
Hour/week, *n*	
Full time	578 (78.9)
Part time	155 (21.1)
Shift profile, *n*	
Shift worker	373 (50.9)
Daily worker	249 (34.0)
Only mornings	107 (14.6)
Only nights	4 (0.5)
Over time work, h	25.6 (23.7–27.6)
Patients care for, last shift, *n*	17.3 (15.8–18.7)
Patients admitted, last shift, *n*	3.3 (2.7–4.0)
Patients discharged, last shift, *n*	2.8 (2.3–3.4)
Model of care delivery, *n*	
Functional	379 (51.7)
Team nursing	255 (34.8)
Other	99 (13.5)
Nursing resources adequacy, 0–100^c^	63.2 (61.5–64.8)
Professional outcomes	
Intention to leave, *n*	
Yes	148 (20.2)
No	553 (75.4)
Missing	32 (4.4)
Role satisfaction, 0–4^a^	2.5 (2.5–2.6)
Satisfaction of being a nurse, 0–4^a^	2.8 (2.8–2.9)
Working group satisfaction, 0–4^a^	2.4 (2.3–2.4)
Non‐nursing tasks	
How often, 0–4^b^	2.5 (2.5–2.6)
When	
Morning	378 (54.5)
Afternoon	148 (21.4)
Night	67 (9.7)
Mornings and afternoons	60 (8.7)
24/24 h	40 (5.8)
Time dedicated, 0–100^c^	32.6 (31.4–33.7)
Non‐nursing tasks reasons, 0–4^d^	
Compensating the lack of resources	2.69 (2.64–2.75)
Being pressed by the organisational culture	2.50 (2.44–2.56)
Dealing unexpected clinical events	2.50 (2.43–2.58)
Protecting patients	2.88 (2.83–2.93)

^a^ From 1, *never*, to 4, *always*.

^b^ From 1, *never*, to 4, *very often*.

^c^ From 5, *none*, to 100%, *the entire shift*.

^d^ From 1, *not a reason*, to 4, *a significant reason*.

* <.05.

** <.01.

In the last shift, participants cared for an average of 17.3 (95% CI 15.8–18.7) patients and managed around three patients discharged and three newly admitted. The nursing care was delivered mainly according to the functional model (379, 51.7%), and at the question, ‘How often nursing resources are adequate in your working context?’ participants ranked adequacy on average 63.2% out of 100 (95% CI 61.5–64.8%).

Participants' satisfaction in the role was on average 2.5 out of 4 (95% CI 2.5–2.6) but was higher for individuals as a nurse (2.8, 95% CI 2.8–2.9) than for the team (2.4, 95% CI 2.3–2.4). Around a quarter of nurses (148, 20.2%) expressed their intention to leave the unit in the next months (Table 1).

### 3.2 Prevalence and factors affecting non‐nursing tasks

Almost all nurses (693, 94.5%) performed at least one type of non‐nursing task (Table 2). These were primarily performed in the mornings (378, 54.5%) and for about 32.6% of the shift time (95% CI 31.4–33.7%). Administrative (531 nurses, 72.4%) and auxiliary (489, 66.7%) tasks were mostly performed, whereas those pertaining to allied health care professionals (187, 25.5%) and medical profiles (136, 18.6%) were performed to a lesser extent. Tasks pertaining to allied health care professionals, although only a few, were performed more often (2.7 mean, 95% CI 2.6–2.8, *p* < .01) and reported as occupying a more significant amount of time (35.2% of the shift, 95% CI 32.8–27.6, *p* < .05) as compared with other forms of non‐nursing tasks (Table 2).

**TABLE 2:** Demographic, professional, perceived reasons and professional outcomes according to non‐nursing tasks performed

	I perform auxiliary tasks		I perform administrative tasks		I perform allied care professionals' task		I perform medical tasks	
Variables	Yes *N* (%), average (95% CI)	No *N* (%), average (95% CI)	Yes *N* (%), average (95% CI)	No *N* (%), average (95% CI)	Yes *N* (%), average (95% CI)	No *N* (%), average (95% CI)	Yes *N* (%), average (95% CI)	No *N* (%), average (95% CI)
Participants, *n*	489 (66.7)	244 (33.3)	531 (72.4)	202 (27.6)	187 (25.5)	546 (74.5)	136 (18.6)	597 (81.4)
Demographic variables								
Gender, *n*								
Female	405 (82.8)	211 (86.5)	444 (83.6)	172 (85.1)	153 (81.8)	463 (84.8)	102 (75.0)**	514 (86.1)
Male	84 (17.2)	33 (13.5)	87 (16.4)	30 (14.9)	34 (18.2)	83 (15.2)	34 (25.0)**	83 (13.9)
Age, years	44.1 (43.3–44.8)*	42.6 (41.3–43.8)	43.7 (43.0–44.5)	43.1 (41.9–44.3)	42.4 (41.1–43.8)*	43.9 (43.2–44.7)	43.6 (42.9–44.2)	43.6 (42.9–44.4)
Professional variables								
Nursing education, *n*								
Nursing Diploma	345 (70.6)*	153 (62.7)	357 (67.2)	141 (69.8)	116 (62.0)	382 (70.0)	94 (69.1)	404 (67.7)
Nursing Bachelor	117 (23.9)*	79 (32.4)	141 (26.6)	55 (27.2)	62 (33.2)	134 (24.5)	38 (27.9)	158 (26.5)
Nursing Diploma + Bachelor	27 (5.5)*	12 (4.9)	33 (6.2)	6 (3.0)	9 (4.8)	30 (5.5)	4 (2.9)	35 (5.9)
Advanced educated, *n*	69 (14.1)	42 (17.2)	86 (16.1)	25 (13.3)	38 (20.3)*	73 (13.3)	24 (17.6)	87 (14.5)
Experience as a nurse, years	22.4 (21.6–23.2)	21.1 (19.7–22.4)	22.1 (21.3–23.0)	21.4 (20.0–22.8)	20.7 (19.2–22.1)*	22.4 (21.6–23.2)	21.5 (19.9–23.1)	22.09 (21.3–22.8)
Experience in the setting, years	12.8 (12.1–13.6)**	10.4 (9.4–11.3)	12.1 (11.4–12.9)	11.7 (10.6–12.9)	11.6 (10.2–12.9)	12.2 (11.4–12.9)	13.3 (11.7–14.8)	11.77 (11.0–12.4)
Working at, *n*								
Hospital	432 (88.3)**	167 (68.4)	435 (81.9)	164 (81.2)	152 (81.3)	447 (81.9)	114 (83.8)	485 (81.2)
Community	23 (4.7)**	36 (14.8)	43 (8.1)	16 (7.9)	12 (6.4)	47 (8.6)	8 (5.9)	51 (8.5)
Residential	22 (4.5)**	29 (11.6)	36 (6.8)	15 (7.4)	14 (7.5)	37 (6.8)	9 (6.6)	42 (7.0)
Free lance	8 (1.6)**	7 (2.9)	11 (2.1)	4 (2.0)	8 (4.3)	7 (1.3)	2 (1.5)	13 (2.2)
Other	4 (0.8)**	5 (2.0)	6 (1.1)	3 (1.5)	1 (0.5)	8 (1.5)	3 (2.2)	6 (1.0)
Setting, *n*								
Medical	157 (32.1)**	72 (29.5)	169 (31.8)	60 (29.7)	63 (33.7)*	166 (30.4)	42 (30.9)*	187 (31.3)
Critical care	104 (21.3)**	50 (20.5)	105 (19.8)	49 (24.3)	44 (23.5)*	110 (20.1)	30 (22.1)*	124 (20.8)
Surgical	110 (22.5)**	24 (9.8)	100 (18.8)	34 (16.8)	39 (20.9)*	95 (17.4)	37 (27.2)*	97 (16.2)
Maternal/paediatrics care	26 (5.3)**	8 (3.3)	27 (5.1)	7 (3.5)	3 (1.4)*	31 (5.7)	5 (3.7)*	29 (4.9)
Home care	28 (5.7)**	41 (16.8)	50 (9.4)	19 (9.4)	14 (7.5)*	5 (10.1)	11 (8.1)*	58 (9.7)
Nursing home	26 (5.3)**	28 (11.5)	37 (7.0)	17 (8.4)	16 (8.6)*	38 (10.0)	8 (5.9)*	46 (7.7)
Other	38 (7.8)**	21 (8.6)	43 (8.1)	16 (7.9)	8 (4.3)*	51 (9.3)	3 (2.2)*	56 (9.4)
Hour/week, *n*								
Full time	390 (79.8)	188 (77.0)	413 (77.8)	165 (81.7)	146 (78.1)	432 (79.1)	110 (80.9)	468 (78.4)
Part time	99 (20.2)	56 (23.0)	118 (22.2)	37 (18.3)	41 (21.9)	114 (20.6)	26 (19.1)	129 (21.6)
Shift profile, *n*								
Shift worker	285 (58.3)**	88 (36.1)	264 (49.7)	109 (54.0)	103 (55.1)	270 (49.5)	82 (60.3)	291 (48.7)
Daily worker	135 (27.6)**	114 (46.7)	187 (35.2)	62 (30.7)	59 (31.6)	190 (34.8)	39 (28.7)	210 (35.2)
Only mornings	66 (13.5)**	41 (16.8)	77 (14.1)	30 (14.9)	23 (12.3)	84 (15.4)	15 (11.0)	92 (15.4)
Only nights	3 (0.3)**	1 (0.4)	3 (0.6)	1 (0.5)	2 (1.1)	2 (0.4)	0	4 (0.7)
Over time work, h	24.4 (22.0–26.7)**	17.8 (15.2–20.4)	22.1 (20.3–24.0)	20.3 (16.9–23.7)	28.6 (24.3–33.0)**	20.1 (18.2–22.0)	27.5 (22.6–32.4)**	20.9 (12.0–22.8)
Patients care for, last shift, *n*	16.62 (14.8–18.3)	18.74 (16.0–21.4)	17.6 (15.8–19.3)	16.4 (13.6–19.3)	19.14 (16.1–22.1)	16.6 (14.9–18.3)	17.2 (14.5–20.0)	17.32 (15.6–19.0)
Patients admitted, last shift, *n*	3.5 (2.7–4.4)	2.9 (1.9–4.0)	3.5 (2.7–4.3)	2.9 (1.9–4.0)	2.4 (1.6–3.2)	3.7 (2.9–4.6)	5.2 (3.2–7.3)**	2.9 (2.2–3.5)
Patients discharged, last shift, *n*	3.0 (2.3–3.7)	2.6 (1.6–3.5)	2.9 (2.2–3.6)	2.8 (1.8–3.9)	3.1 (2.0–4.2)	2.8 (2.1–3.5)	4.3 (2.3–6.3)	2.5 (2.0–3.0)
Model of care delivery, *n*								
Functional	257 (52.6)**	122 (50.0)	273 (51.4)	106 (52.5)	108 (57.8)	271 (49.6)	72 (52.9)	307 (51.4)
Team nursing	182 (37.2)**	73 (30.0)	186 (35.0)	69 (34.2)	60 (32.1)	195 (35.7)	53 (39.0)	202 (33.9)
Other	50 (10.2)**	49 (20.0)	72 (13.6)	27 (13.3)	19 (10.1)	80 (14.6)	11 (8.1)	88 (14.7)
Nursing resources adequacy, 0–100^c^	61.0 (58.9–63.0)**	67.8 (64.8–70.8)	61.6 (59.6–63.6)**	67.6 (64.4–70.9)	56.9 (53.6–60.2)**	65.4 (63.5–67.3)	57.5 (53.2–61.8)**	64.5 (62.6–66.3)
Professional outcomes								
Intention to leave, *n*								
Yes	105 (21.5)	43 (17.6)	110 (20.7)	38 (18.8)	45 (23.8)	103 (18.8)	31 (22.7)	117 (19.6)
No	366 (74.8)	187 (76.6)	399 (75.1)	144 (71.2)	136 (72.7)	417 (76.4)	99 (72.7)	474 (79.4)
Missing	18 (3.7)	14 (5.8)	22 (4.2)	20 (9.9)	8 (4.2)	26 (4.8)	6 (4.4)	6 (1.0)
Role satisfaction, 0–4^a^	2.5 (2.4–2.6)	2.6 (2.5–2.7)	2.5 (2.5–2.6)	2.6 (2.5–2.7)	2.5 (2.3–2.6)	2.6 (2.5–2.6)	2.54 (2.3–2.6)	2.60 (2.5–2.6)
Satisfaction of being a nurse, 0–4^a^	2.8 (2.7–2.9)	2.9 (2.7–3.0)	2.8 (2.7–2.9)	2.8 (2.7–3.0)	2.9 (2.7–3.0)	2.8 (2.7–2.9)	2.8 (2.6–2.9)	2.88 (2.8–2.9)
Working group satisfaction, 0–4^a^	2.4 (2.3–2.4)	2.4 (2.3–2.5)	2.4 (2.3–2.5)	2.4 (2.2–2.5)	2.3 (2.2–2.5)	2.4 (2.3–2.5)	2.3 (2.2–2.5)	2.43 (2.3–2.5)
Non‐nursing tasks								
How often, 0–4^b^	2.6 (2.5–2.6)	‐	2.6 (2.5–2.6)	‐	2.7 (2.6–2.8)*	‐	2.6 (2.5–2.7)	‐
When								
Morning	252 (51.5)	‐	291 (54.8)	‐	97 (51.9)	‐	64 (47.1)	‐
Afternoon	106 (21.7)	‐	107 (20.2)	‐	43 (23.0)	‐	26 (19.1)	‐
Night	55 (11.2)	‐	52 (9.8)	‐	12 (6.4)	‐	21 (15.4)	‐
Mornings and afternoons	43 (8.8)	‐	51 (9.6)	‐	16 (8.6)	‐	11 (8.1)	‐
24/24 h	33 (6.7)	‐	30 (5.6)	‐	19 (10.2)	‐	14 (10.3)	‐
Time dedicated, 0–100^c^	33.05 (31.6–34.4)	‐	33.0 (31.6–34.3)	‐	35.2 (32.8–37.6)*	‐	33.5 (30.7–36.3)	‐
Non‐nursing tasks reasons, 0–4^d^								
Compensating the lack of resources	2.71 (2.64–2.78)	2.66 (2.56–2.76)	2.69 (2.62–2.76)	2.70 (2.59–2.81)	2.78 (2.67–2.89)	2.66 (2.59–2.73)	2.69 (2.55–2.83)	2.70 (2.63–2.76)
Being pressed by the organisational culture	2.35 (2.28–2.42)	2.40 (2.30–2.50)	2.37 (2.30–2.44)	2.35 (2.24–2.46)	2.35 (2.24–2.46)	2.73 (2.30–2.44)	2.39 (2.24–2.53)	2.36 (2.30–2.42)
Dealing unexpected clinical events	2.49 (2.40–2.58)	2.53 (2.41–2.64)	2.53 (2.45–2.61)	2.41 (2.26–2.56)	2.56 (2.44–2.68)	2.48 (2.39–2.57)	2.73 (2.59–2.88)**	2.44 (2.36–2.52)
Protecting patients	2.87 (2.81–2.94)	2.94 (2.85–3.03)	2.90 (2.85–2.96)	2.85 (2.75–2.95)	2.89 (2.80–2.97)	2.89 (2.83–2.95)	2.95 (2.84–2.94)	2.88 (2.82–2.93)

^a^ From 1, *never*, to 4, *always*.

^b^ From 1, *never*, to 4, *very often*.

^c^ From 5, *none*, to 100%, *the entire shift*.

^d^ From 1, *not a reason*, to 4, *a significant reason*.

* <.05.

** <.01.

As reported in Table 2, auxiliary tasks were performed by older nurses (44.1 vs. 42.6 years, *p* < .05) with more experience in the setting (12.8 vs. 10.4 years, *p* < .01) and with a nursing diploma (70.6% vs. 62.7%, *p* < .05). They were performed mainly by nurses working in a hospital (88.3 vs. 68.4%, *p* < .01) and as shift workers (58.3 vs. 36.1%, *p* < .01). Furthermore, nurses who carried out auxiliary tasks reported on average more overtime work in the last 3 months (24.4 vs. 17.8 h, *p* < .01), a lower adequacy of nursing resources (61.0% vs. 67.8% of the shift time, *p* < .01) and a greater likelihood to work according to the functional model (52.6 vs. 50.0%, *p* < .01) as compared with nurses who did not perform auxiliary tasks.

Nurses performing administrative tasks reported a lower perception of resource adequacy than nurses who did not perform administrative duties (61.6 vs. 67.6%, *p* < .01). Instead, nurses who performed allied health care professionals' tasks were younger (42.4 vs. 43.9 years, *p* < .05), more often advanced educated (20.3 vs. 13.3%, *p* < .05), with less experience as a nurse (20.7 vs. 22.4 years, *p* < .05), working more often in medical settings (33.7% vs. 30.4%, *p* < .05) and reported more overtime work (28.6 vs. 20.1 h, *p* < .01) and a lower adequacy of nursing resources (56.9% vs. 65.4% of the shift, *p* < .01) as compared with those who did not perform tasks of allied health care professionals.

Medical tasks have been reported to be performed more often by male nurses (25 vs. 13.9%, *p* < .01) and by nurses working in surgical (27.2 vs. 16.2%, *p* < .05) and in critical care settings (22.1 vs. 20.8%, *p* < .05). Nurses who performed these tasks reported a higher average of overtime working hours (27.5 vs. 20.9 h, *p* < .01), of patients admitted in the last shift (5.2 vs. 2.9, *p* < .01) and a lower adequacy of nursing resources (57.5 vs. 64.5%, *p* < .01). Nurses who performed medical tasks reported the occurrence of higher unexpected clinical events as compared with those who did not perform these tasks (2.73 mean vs. 2.44, *p* < .01). No other statistical differences have emerged, as reported in Table 2.

### 3.3 The path analysis

**FIGURE 1:**
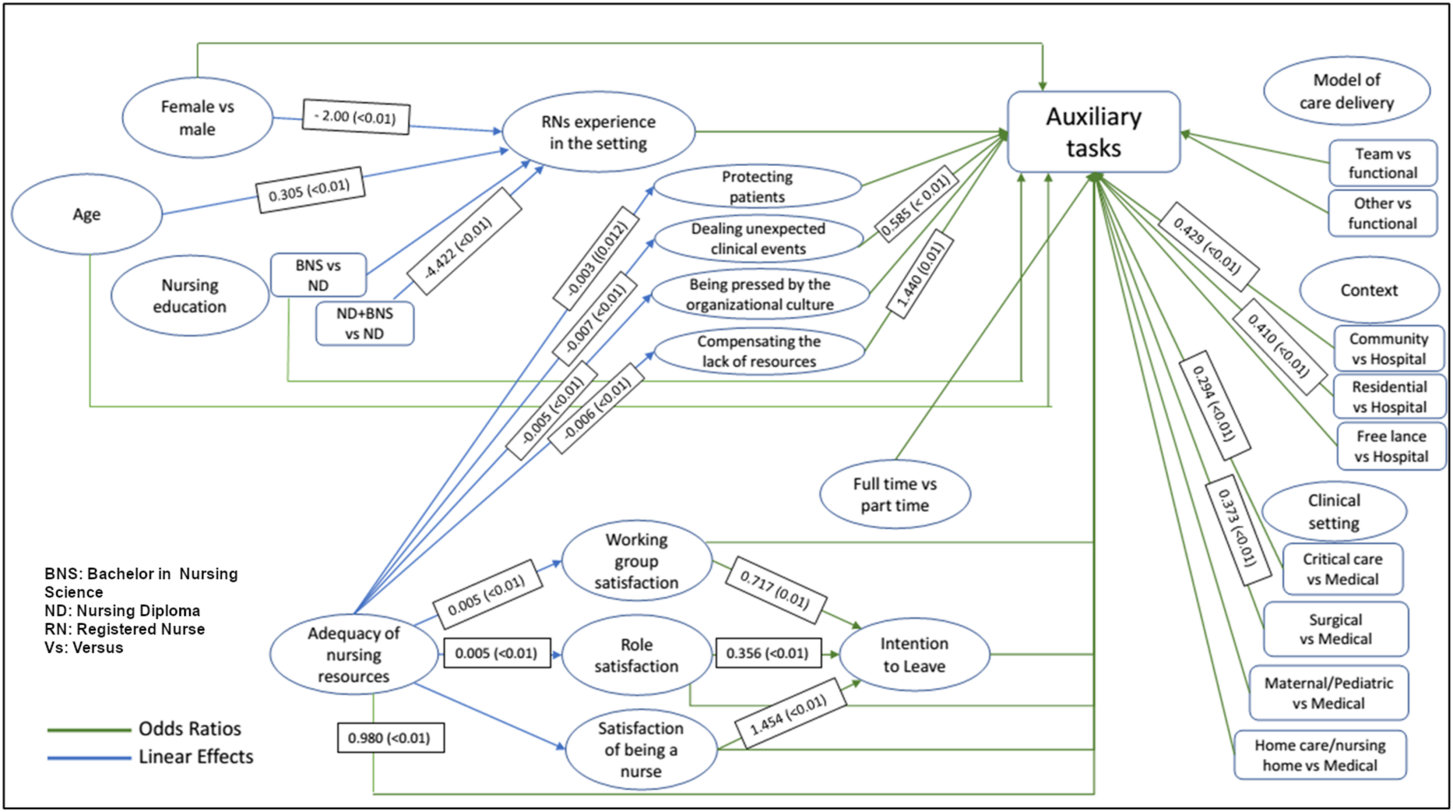
Path analysis

As shown by Figure 1 and Table 3, working in community (OR 0.43, 95% CI 0.29–0.63, *p* < .01) or in residential (OR 0.41, 95% CI 0.23–0.72, *p* < .01) settings, as compared with working in a hospital, reduced the likelihood of performing auxiliary tasks and of working in surgical (OR 0.37, 95% CI 0.19–0.75, *p* < .01) or in critical (OR 0.29, 95% CI 0.16–0.54, *p* < .01) settings rather than in medical settings. Greater adequacy of nursing resources slightly decreased (OR 0.98, 95% CI 0.97–0.99, *p* < .01) the likelihood of performing auxiliary tasks. The more nurses perceived the need to compensate for the lack of resources, the higher the likelihood of performing auxiliary tasks (OR 1.44, 95% CI 1.07–1.93, *p* < .01), and when nurses were called to deal with unexpected clinical events, the likelihood to perform auxiliary tasks decreased (OR 0.58, 95% CI 0.44–0.77, *p* < .01).

**TABLE 3:** Path analysis coefficients

Non‐nursing auxiliary tasks	*β*/OR [CI 95%]	*p* value
Teams vs. functional model	1.267 [0.648–2.477]	.490
Other model vs. functional	1.422 [0.706–2.865]	.324
Working group satisfaction	0.977 [0.775–1.232]	.844
Role satisfaction	1.071 [0.815–1.409]	.621
Satisfaction of being a nurse	1.136 [0.906–1.423]	.269
Intention to leave	1.122 [0.703–1.792]	.629
Adequacy of nurses' resources	0.980 [0.972–0.988]	<.01
Compensating the lack of resources	1.440 [1.072–1.933]	.015
Being pressed by the organisational culture	0.917 [0.712–1.18]	.500
Dealing with unexpected clinical events	0.585 [0.445–0.768]	<.01
Protecting patients	0.966 [0.718–1.298]	.817
Working hours/week	0.868 [0.563–1.339]	.522
Community vs. hospital	0.429 [0.290–0.634]	<.01
Residential care vs. hospital	0.410 [0.232–0.722]	.002
Freelance vs. hospital	2.218 [0.177–27.803]	.537
Critical care vs. medical	0.294 [0.16–0.538]	<.01
Surgical vs. medical	0.373 [0.187–0.746]	.005
Maternal/paediatric vs. medical	0.657 [0.195–2.215]	.498
Home care/nursing home vs. medical	0.357 [0.088–1.45]	.150
Female vs. male	0.895 [0.550–1.455]	.654
Age	0.996 [0.966–1.027]	.804
Bachelor of Nursing Diploma vs. Nursing Diploma	1.134 [0.501–2.567]	.763
Nursing Diploma + Bachelor vs. Nursing Diploma	0.627 [0.355–1.109]	.109
Experience as RN in the unit	1.022 [0.997–1.044]	.085
_cons	17.492 [1.703–179.629]	.016
Experience as RN in the unit		
Female vs. male	−2.008 [−3.51 to −0.506]	.009
Age	0.305 [0.210–.400]	<.01
Bachelor of Nursing Diploma vs. Nursing Diploma	−2.161 [−4.837 to 0.516]	.114
Nursing Diploma + Bachelor vs. Nursing Diploma	−4.422 [−6.219 to −2.625]	<.01
_cons	1.853 [−2.861 to 6.568]	.441
Compensating the lack of resources		
Adequacy of nurses' resources	−0.006 [−0.008 to −0.003]	<.01
_cons	3.016 [2.868–3.165]	<.01
Being pressed by the organisational culture		
Adequacy of nurses' resources	−0.005 [−0.007 to −0.003]	<.01
_cons	2.73 [2.575–2.885]	<.01
Dealing with unexpected clinical events		
Adequacy of nurses' resources	−0.007 [−0.01 to −0.005]	<.01
_cons	2.835 [2.675–2.995]	<.01
Protecting patients		
Adequacy of nurses' resources	−0.003 [−0.005 to −0.001]	.012
_cons	3.012 [2.875–3.15]	<.01
Working group satisfaction		
Adequacy of nurses' resources	0.006 [0.003–0.008]	<.01
_cons	2.09 [1.903–2.277]	<.01
Role satisfaction		
Adequacy of nurses' resources	0.005 [0.003–0.008]	<.01
_cons	2.25 [2.076–2.423]	<.01
Satisfaction of being a nurse		
Adequacy of nurses' resources	0.001 [−0.002 to 0.004]	.517
_cons	2.812 [2.625–3.000]	<.01
Intention to leave		
Working group satisfaction	0.717 [0.557–0.924]	<.01
Role satisfaction	0.356 [0.266–0.477]	<.01
Satisfaction of being a nurse	1.454 [1.148–1.841]	.002
_cons	2.441 [0.165–1.619]	.016

Abbreviations: *β*, beta coefficient; _cons, constant; CI, confidence interval; OR, odds ratio; RN, registered nurse; vs., versus.

With regard to indirect effects—as variables affecting those illustrated in Figure 1—being female (*β =* −2.00, 95% CI −3.51 to −0.50, *p* < .01), compared with male, and having a Nursing Diploma plus a Bachelor in Nursing (*β* = −4.42, 95% CI −6.21 to −2.62, *p* < .01) as compared with having only a Nursing Diploma reduced the RNs experience in the setting, which was, on the other hand, increased by age (*β* = .30, 95% CI 0.21–0.40, *p* < .01). However, the experience in the setting did not significantly affect whether or not auxiliary tasks were performed.

Moreover, the adequacy of nursing resources reported small indirect effects by reducing reasons for non‐nursing tasks (‘Compensating the lack of resources’ *β* = −.006, 95% CI −0.008 to −0.003; ‘Being pressed by the organisational culture’ *β =* −.005, 95% CI −0.007 to −0.003; ‘Dealing unexpected clinical events’ *β* = −.007, 95% CI −0.01 to −0.005, *p* < .01; ‘Protecting patients’ *β* = −.003, 95% CI −0.005 to −0.001, *p* = .012) and by increasing satisfaction on working group and role (*β* = .006, 95% CI 0.003–0.008; *β* = 0.005, 95% CI 0.003–0.008, *p* < .01). These, in turn, had indirect effects by reducing intention to leave (working group satisfaction OR 0.71, 95% CI 0.56–0.92 and role satisfaction OR 0.72, 95% CI 0.27–0.48, *p* < .01, respectively), which was instead increased by the satisfaction of ‘Being a nurse’ (OR 1.45, 95% CI 1.15–1.84, *p* < .01). However, the nurse's intention to leave and satisfaction did not significantly affect whether or not auxiliary tasks were performed.

## 4 DISCUSSION

To our best knowledge, this is the first large study including an entire community of nurses belonging to the same geographical area, registered in the same Nursing Board and working in the same geographical context, thus sharing similar professional experiences and culture. Advancing the knowledge on non‐nursing tasks and understanding factors involved in a large context might help policymakers to shape appropriate interventions to minimize its occurrence and increase the value of nursing time.

### 4.1 Participants

We involved all active nurses, and the majority of them participated: the response rate was in line with that documented in other surveys performed among nurses (e.g., VanGeest & Johnson, 2011). Participants were mainly female, in a mature age (mean 43.6 years old) and with a long experience in hospital care, also in line with the profile of nurses documented at the national level (Grosso et al., 2019). Although the data should be interpreted according to contexts, as hospital or community settings, on average, nurses reported to have cared for in the last shift around 17 patients accompanied by three newly admitted and three discharged patients: these data confirm the unfavourable nurse‐to‐patient ratio (FNOPI, 2018) already reported in the Italian context.

### 4.2 Prevalence and variables affecting non‐nursing tasks

Nurses reported dedicating one‐third of their shift time to non‐nursing tasks: only few (5.5%) documented to spend their shift entirely to perform interventions falling within the scope of their discipline. Consequently, the amount of care planned for around 17 patients for each nurse, which should be considered really critical, is further eroded due to the time spent in tasks that other professionals should perform.

Participants perform, in order, mainly administrative (72.4%) and auxiliary (66.7%) tasks as documented across 12 countries where >90% of nurses have been reported to perform these kinds of tasks (Bruyneel et al., 2013). Differently, the occurrence of administrative tasks has been documented to a lesser extent in previous studies, namely, from 7% (Westbrook et al., 2011) to 35% (Hendrich et al., 2008) of the working time. This difference might be interpreted under different lines: (a) as a consequence of a different concept of administrative tasks—whether expressed as the substitution of the secretary role or only those activities connected with patients' care (Hendrich et al., 2008); (b) as an expression of the increased bureaucratization of the care processes requiring additional personnel; and/or (c) as an expression of the lack of resources supporting the units (e.g., secretaries) due to the rationed measures applied to health care services.

On the other hand, only a quarter of nurses performed tasks belonging to allied health care professionals, likely because they are less traceable given that they might express forms of interprofessional teamwork (Grosso et al., 2019). Moreover, less than 20% of nurses reported performing medical tasks, in line with the evidence available (from 24% to 29.2%, in six countries; Maier et al., 2018). Therefore, nurses seem to be less involved in tasks of allied health care and medical professions, while they are more often called to perform tasks that can be delegable to nurses' aides, assistants, unlicensed health workers and administrative staff.

Only some factors have emerged as significantly different across groups, and in some cases (e.g., see age), the difference seems to have limited practical meaning. However, some individual (higher age) and professional (being vocationally educated with a diploma, higher experience in the setting) variables seem to expose nurses to the risk of performing auxiliary tasks. In contrast, male nurses seem more likely to perform medical tasks. Moreover, some variables at the organisational levels, such as the context (hospital, nursing home) and the models of care delivery (functional model), seem to engage nurses in performing auxiliary tasks, while the increased number of patients admitted and their critical condition seems to trigger medical tasks. Furthermore, when nurses perceive more nursing resources, they seem to be more engaged in auxiliary and administrative tasks, but when their perception is worse, they seem to perform more medical tasks than other health care professionals. Some of these factors express attitudes shaped during education and clinical experience (e.g., being flexible) and are modifiable by appropriate organisational interventions (Palese, Ambrosi, et al., 2019). However, doing non‐nursing tasks seems to be not affected by the degree of satisfaction and by the intention to leave, which is similar across groups, suggesting that doing a non‐nursing task is normalized in a sort of ‘pragmatic acceptance’ (Gibbon & Crane, 2018).

### 4.3 The path analysis

In the path analysis, where several indirect and direct explanatory variables have been introduced, a few factors have emerged as influencing the occurrence of auxiliary task by reporting minor effects, suggesting that the phenomenon is multifactorial and at merit for further studies. First, nurses working in hospital and in medical settings and those who perceive the need to compensate for the lack of resources at the unit level are more exposed to the risk of performing auxiliary tasks, as documented previously (e.g., Bruyneel et al., 2013) suggesting that units should also be equipped with auxiliaries. On the other hand, those who frequently deal with unexpected clinical events are less likely to perform auxiliary tasks because they are concentrated on the patient's clinical condition as a priority. Therefore, the setting, with its clinical mission, resources and culture, seems to have a relevant role on auxiliary tasks occurrence, highlighting the need of having a clear and agreed job description capable of reflecting the peculiarity of the context and preventing activities wasting nurses' time.

Second, the perception of adequacy in nursing resources emerged as a factor affecting several variables, albeit with a small effect. Perceiving adequate nursing staffing prevents some non‐nursing tasks and increases the working group and role satisfaction. These prevented the intention to leave that was otherwise increased by a higher satisfaction of being a nurse. Despite these effects, intention to leave did not affect the likelihood of performing auxiliary tasks. Moreover, being pressured by the organisational culture and protecting patients (e.g., Bruyneel et al., 2013; Grosso et al., 2019) did not report any association with auxiliary task, and this suggests that doing these tasks is considered ‘normal’ by nurses (Gibbon & Crane, 2018), as a part of their routine.

### 4.4 Limitations

This study has several limitations. First, we adopted a cross‐sectional design where non‐nursing tasks and their explanatory variables were collected simultaneously, requiring caution in considering factors that emerged as causal. Second, nurses were provided with some examples of non‐nursing tasks to uniform the interpretation of each category; however, their personal conceptions regarding the non‐nursing tasks and what nursing care is might have introduced some biases. For example, performing venepuncture has been defined as non‐nursing activities in some studies (e.g., Bruyneel et al., 2013), whereas, in our context, these are considered nursing tasks. Third, participants were required to indicate none, one or more non‐nursing task categories, according to what they did in their last shift, and bias might have influenced the precision in the time spent by them in each task. However, the aim was to explore the issue and not to document precisely the amount of time spent in each non‐nursing task, a finding that might be explored with other instruments (e.g., time and motion analysis) (Desjardins et al., 2008).

Fourth, we collected data with two main procedures, via paper/pencil and e‐mail address, in order to maximize the participation rate; however, this decision might have introduced both selection and information biases. Additionally, we included nurses working in different settings (hospital and community) where some issues might have been addressed differently (e.g., paying or not the nurses' overtime) without performing any stratification of the data (e.g., the number of patients cared for). However, the exploratory nature of the study was to describe a global picture; future studies might deepen the profile of nurses in each setting in order to develop contextualized evidence.

## 5 CONCLUSION

In a large mountain province, only a few registered nurses perform only nursing tasks. The large majority perform administrative and auxiliary tasks, whereas medical tasks and that of allied health care professionals are performed with less frequency. Around one‐third of the shift time is spent doing other tasks rather than nursing care, suggesting that in conditions with a poor nurse‐to‐patient ratio as documented in this study, nursing care might be further eroded by the time devoted to non‐nursing tasks. The number of auxiliary tasks, which express a clear waste of nursing time, is high in hospital settings, where units are poorly supported with nursing and auxiliary staff and where patients with predictable clinical issues are cared for.

## 6 IMPLICATIONS FOR NURSING MANAGEMENT

Strategies to increase the time available for nursing care should consider the type of tasks performed by nurses and their antecedents. The focus of nurse managers should be on tasks implying a clear waste of nursing time and not adding value to care rather than those that might improve the quality of the overall care and benefit the patient. In fact, in a wider perspective, even though each activity might be perceived as a waste of time by nurse managers and nurses, it does not mean that this really is a waste of time if it is still a value‐added care. In order to identify interventions, nurse managers might assess activities by considering if these (a) increase the time wasted, thus eroding time available for nursing care; (b) require less education and/or competences, thus wasting the nursing education investment; and (c) are nonvalue‐added activity, thus are not capable to produce benefit on patients. Beyond the recognized consequences on patients (missed nursing care) and nurses (e.g., dissatisfaction), these activities are not cost‐effective and require to be reallocated to increase care effectiveness, ward productivity and efficiency.

However, identifying what objectively is a task wasting time, wasting the education investments or not benefiting the patients might not be a straightforward process. Thus, several steps should be systematically put into practice.

First, tasks performed daily by nurses and embodied in their routine should be appropriately traced, for example, with a day‐index collection of data via observation, allowing their detection also in the time devoted to them. In order to promote a shared meaning and action in each specific context, nurses should be involved in interpreting this data through audits and/or focus groups. Data collection and discussion at the unit level might also allow the full consideration of the clinical complexity of patients in that context and the availability of other health care professional or auxiliary/nurse's aide resources.

Second, it might be important to assess the quality and the appropriateness of the delegation skills possessed by nurses and consequently to coach them in improving such skills. The Italian nursing profession is ending its transition from a vocational to a university education; those nurses educated in the nursing diploma might have been trained to perform all activities required by patients and units. Therefore, alongside the availability of an appropriate support staff, they might require to develop delegation skills to protect their time in favour of those activities requiring nursing competences and expertise.

On the other hand, a discussion on the concept of non‐nursing tasks is at a merit of consideration in the context of the transitions lived by the nursing profession. With increased education, some fundamental care needs may be considered as non‐nursing care, thus out of the scope of the nursing discipline and at need to be performed by another professional as nurses' aides, assistants or lay health workers. Collecting and discussing examples of non‐nursing activities to assess the real nature of tasks performed on a daily basis by nurses, as for example expression of the care offered towards fundamental needs, might be useful.

Searching for different points of view to understand the phenomenon might help nurse managers to design interventions to increase the value of nursing time. Furthermore, engaging nurses in finding solutions might also help them to prevent stress, frustration, feelings and dissatisfaction and to promote proactive approaches. For example, administrative tasks can be managed by implementing electronic health records or by revising the documentation process in an innovative way.

However, continuing to study the reasons for non‐nursing tasks in the work environment, according to the limited contribution of factors discovered to date, might increase awareness and help in designing interventions to prevent task confusion and shifting, nonvalue‐added care and, ultimately, issues in cost‐effective models of care.

## CONFLICT OF INTEREST

No conflict of interest has been declared by the authors.

## ETHICAL STATEMENT

According to the nature of the study, which was based upon a survey, no ethical approval was required.

## AUTHOR CONTRIBUTIONS

SG, ST, IB, CJ, DDM, LD, GF, ML, NO, LPDM and AP made substantial contributions to conception and design or acquisition of data. SG, AP, JL and LG made substantial contributions to analysis and interpretation of data and involved in drafting the manuscript or revising it critically for important intellectual content. SG, JL, ST, IB, CJ, DDM, LD, GF, ML, NO, LG, LPDM and AP gave final approval of the version to be published. Each author should have participated sufficiently in the work to take public responsibility for appropriate portions of the content. SG, JL, ST, IB, CJ, DDM, LD, GF, ML, NO, LG, LPDM and AP agreed to be accountable for all aspects of the work in ensuring that questions related to the accuracy or integrity of any part of the work are appropriately investigated and resolved.

## Supporting information


**Table S1.** Strengthening the Reporting of Observational studies in Epidemiology (STROBE) Checklist in reports of cross‐sectional studies.

**Table S2.** Exploratory Factorial Analysis (EFA): reasons of non‐nursing tasks

## Data Availability

Data available on request from the authors
