# Supplementary material for: Prevalence and reasons for non‐nursing tasks as perceived by nurses: Findings from a large cross‐sectional study
Source: J Nurs Manag. 2021 Aug 31;29(8):2658–73. doi: 10.1111/jonm.13451 (PMC9291208; doi:10.1111/jonm.13451)
Supplement: Supplementary file 2 — Table S2. Exploratory Factorial Analysis (EFA): reasons of non‐nursing tasks [file JONM-29-2658-s002.docx]

- 1. **Supplementary Table 2. Exploratory Factorial Analysis (EFA): reasons of non-nursing tasks**

|  | F1 | F2 | F3 | | F4 |  |
| --- | --- | --- | --- | --- | --- | --- |
| **Items** | **Compensating the lack of resources** | **Being pressed by the organisational culture** | **Dealing with unexpected clinical events** | | **Protecting patients** | Average (95% CI)§ |
| 2. Lack in nursing care | .789 |  |  | |  | 2.79 (2.72-2.86) |
| 12. Insufficient number of human resources supporting the unit | .738 |  |  | |  | 2.98 (2.91-3.04) |
| 3. Inadequate nurse-to-patient ratio | .697 |  |  | |  | 2.65 (2.57-2.72) |
| 13. Lack of nurses’ aides | .621 |  |  | |  | 2.89 (2.81-2.96) |
| 7. Excessive workloads | .527 |  |  | |  | 2.64 (2.58-2.70) |
| **% Explained variance** | **20.78** |  |  | |  | **2.69 (2.64 - 2.75)** |
| **Cronbach’s Alpha** | **.796** |  |  | |  |  |
| 15. Organisational routine |  | .825 |  | |  | 2.56 (2.49-2.64) |
| 16. Rules (formally or informally) established by other health care professionals |  | .792 |  | |  | 2.42 (2.36-2.49) |
| 17. Rules (formally or informally) established by the headquarters of the department/hospital |  | .774 |  | |  | 2.56 (2.49-2.63) |
| **% Explained variance** |  | **17.633** |  | |  | **2.50 (2.44 -2.56)** |
| **Cronbach’s Alpha** |  | **.811** |  | |  |  |
| 5. Unexpected critical patients/situations |  |  | .882 | |  | 2.50 (2.43-2.56) |
| 6. Unexpected number of critical patients |  |  | .860 | |  | 2.44 (2.38-2.51) |
| 4. High number of admission/resignations |  |  | .595 | |  | 2.45 (2.37-2.52) |
| **% Explained variance** |  |  | 15.000 | |  | 2.50 (2.43 - 2.58) |
| **Cronbach’s Alpha** |  |  | **.767** | |  |  |
| 10. Ensure patients’ outcomes |  |  |  | | .796 | 3.00 (2.94-3.07) |
| 11. Keep good atmosphere in the team |  |  |  | | .779 | 2.84 (2.78-2.91) |
| 8. Ensure that all tasks required are carried out |  |  |  | | .684 | 3.12 (3.04-3.18) |
| **% Explained variance** |  |  |  | | 13.611 | 2.88 (2.83 - 2.93) |
| **Cronbach’s Alpha** |  |  |  | | .728 |  |
| **Total** |  |  |  | | **67.027** | **2.67 (2.61-2.73)** |
| **Cronbach’s Alpha** |  |  |  | | **.867** |  |
| § Likert scale = 1, not a reason, to 4, a significant reason  Extraction method: analysis of the main component; Rotation method: Varimax with Kaiser standardisation | | | |  |  |  |
| a. Convergence for rotation performed in 6 interactions | | | |  |  |  |

KMO Test = 0.850; Bartlett Chi-Square 1383.221; p <0.000

**Legend.** CI, Confidence Interval; F, Factor; KMO, Kaiser-Meyer-Olkin
